# Editorial: The Application of Radiomics and Artificial Intelligence in Cancer Imaging

**DOI:** 10.3389/fonc.2022.864940

**Published:** 2022-03-02

**Authors:** Hong Huang

**Affiliations:** Key Laboratory of Optoelectronic Technology and Systems, Ministry of Education, Chongqing University, Chongqing, China

**Keywords:** cancer imaging, radiomics, artificial intelligence, deep learning, convolutional neural networks (CNN)

## Introduction

Cancer provides a unique medical decision context considering its variegated forms with the evolution of the disease, as well as the individual condition of patients, their ability to receive treatment, and their responses to treatment. Technological advances in medical imaging bring benefits to address the challenges of accurate detection, characterization, and monitoring of cancer, but traditional imaging assessment of cancer commonly relies on visual evaluations, the interpretations of which may be augmented by advanced computational analyses. Radiomics and artificial intelligence (AI) promises to make great strides in the qualitative and quantitative interpretation of cancer imaging. Radiomics, the high-throughput mining of quantitative image features from standard-of-care medical imaging, is gaining significant importance in cancer research, while AI excels at distinguishing complex patterns in cancer images and thus provides the opportunity to alter image interpretation from a purely qualitative and subjective task to one that can be quantified and effortlessly reproduced. Therefore, this Research Topic recruited studies that explore the application of radiomics and AI in cancer imaging.

We are so glad to see that many wonderful works were submitted to our Research Topic. In the end, a total of 45 papers were published, and all papers were original studies. The studies were conducted in various countries, including China, the USA, Italy, Australia, Spain, Germany, and the Netherlands. The authors explored different methods to explore the role of radiomics and AI in cancer imaging.

## Papers Included in This Research Topic

### Studies With Radiomics in Cancer Imaging

Radiomics aims to extract mineable high-dimensional imaging features from medical images, and it enables data to be extracted and applied within clinical-decision support systems to improve the accuracy of cancer diagnosis, prognosis, and prediction (Shan et al.; Shi et al.; Gao et al.). This type of studies included in the Research Topic used features extracted from different kinds of images, Positron Emission Tomography–Computed Tomography (PET/CT), CT, Magnetic Resonance Imaging (MRI), and Contrast-Enhanced Spectral Mammography, to enhance the performance of differential diagnosis, preoperative differentiation, and preoperative prediction (Wang et al.; Yan et al.; Lin et al.; Wang et al.). Ren et al. assessed the predictive ability of CT-based radiomics signature in the differential diagnosis between pancreatic adenosquamous carcinoma (PASC) and pancreatic ductal adenocarcinoma (PDAC). Chen et al. investigated the role of CT radiomics features combined with a support vector machine (SVM) model in potentially differentiating pelvic rhabdomyosarcoma (RMS) from yolk sac tumors (YSTs) in children. Wu et al. developed a radiomics nomogram for identifying sub-1 cm benign and malignant thyroid lesions. Zhang et al. designed a MRI radiomics-based nomogram for discriminating histological grades 1 and 2 from grade 3 endometrial carcinoma. Hou et al. proposed a radiomics predictive model based on multi-parameter MR imaging features and clinical features to predict lymph node metastasis (LNM) in patients with cervical cancer. Zhang et al. developed a new prognostic biomarker for predicting survival outcomes in breast cancer patients with residual tumors after neoadjuvant chemotherapy. Zhao et al. designed a contrast-enhanced MRI (CE-MRI) radiomics for pretherapeutic prediction of the response to transarterial chemoembolization (TACE) in patients with hepatocellular carcinoma (HCC). Tang et al. demonstrated that radiomics features obtained from nephrographic phase had stronger predictive ability than features from corticomedullary or unenhanced phase and multi-omics models combining radiomics and transcriptome data could further increase the predictive accuracy. Chiloiro et al. developed radiomics features using pre- and post-neoadjuvant chemoradiation (nCRT) MRI for predicting 2 years distant metastasis (2yDM) rate in LARC patients.

### Studies With AI in Cancer Imaging

In general, AI in cancer imaging can be applied two ways: 1) radiomic features extracted from Region-of-Interests (ROIs) can be input into Machine learning methods for subsequent tasks, 2) an entire medical image or image series can be an input into Deep learning (DL) model to perform detection, characterizing, and monitoring of cancers. Zhang et al. developed deep learning-based radiomics signatures based on the B-mode US (B-US-RS) or SWE (SWE-RS) to improve the diagnostic performance in classifying breast masses. Zhu et al. constructed a DL model to predict good responders by training apparent diffusion coefficient (ADC) images from different scanners, and DL-based model is beneficial to reveal the potential of pretreatment apparent diffusion coefficient images for the prediction of good responders to neoadjuvant chemoradiotherapy. Amarasinghe et al. presented an ensemble model of 2.5D convolutional neural networks (CNNs) to automatically segment skeletal muscle on low-quality CTs acquired in PET/CT studies, which can be used to delineate skeletal muscle area at the L3 region of attenuation correction CT scans for patients with non-small cell lung cancer (NSCLC). Huang et al. applied a two-stage Res3DUnet to fully automate the segmentation of the anterior mediastinal lesions from ordinary CT images. Zhou et al. proposed a framework based on hierarchical CNNs for automatic detection and classification of focal liver lesions (FLLs) in multi-phasic CT. Zhou et al. designed a deep learning approach based on contrast-enhanced MR and 3D CNN to predict the microvascular invasion (MVI) in HCC patients. Ran et al. proposed a prediction model based on radiomics signature, deep learning signature, and CT-reported LN status that can be used to predict preoperative lymph node (LN) metastasis in patients with lung adenocarcinoma (LUAD). In this paper, radiomics method and DL method are compared, it is clear that they have their own advantages and disadvantages, so the combination of radiomics features and deep features will bring benefits for cancer imaging.

### Other Related Studies

Furthermore, there are some related works focusing on the application of machine learning methods to improve the results of medical image processing. Wang et al. conducted the Rician noise removal from 3D MR volumetric data through a modified higher-order singular value decomposition (MHOSVD) method. Guan et al. employed imaging quantification and machine learning to discriminate non-calcified hamartoma from adenocarcinoma. Xie et al. developed texture features and machine learning-based analysis of ADC maps for the prediction of Grade Group upgrading in Gleason.

## Conclusion

In conclusion, radiomic features have the potential to obtain biological and pathophysiological information from ROIs, and the corresponding quantitative features can provide rapid and accurate non-invasive biomarkers for cancer diagnostics, prognosis, and treatment response monitoring. AI has got a lot of attention, especially, for the success of deep learning to create complex neural architectures to solve difficult problems which would be impossible with traditional machine learning methods, and AI-based methods have shown significant progress in the field of radiological-based medical imaging applications.

However, the application of AI-based methods in cancer imaging to date has not been vigorously validated for reproducibility and generalizability, there are many challenges that still remain: 1) A large number of labeled images are needed to build generalizable robust AI models, while it is time-consuming to annotate large-scale medical image datasets like ImageNet dataset. To tackle this issue, data augmentation can be used to increase the amount of data by warping, rotating, or inverting existing images, or creating synthetic data from existing date using Generative Adversarial Networks (GANs). Another widely used approach is transfer learning, and it has been established as one of the most practical paradigms in medical image processing with insufficient training samples since it makes full use of the parameters of model pre-trained from large-scale natural image datasets. Due to the big inter-domain discrepancies between natural images and medical images, self-supervised learning provides one possible solution to overcome the limitations of transfer learning, and it learns representations in a self-supervised way which is beneficial to medical image processing. 2) The black-box nature of DL methods is one of the largest stumbling blocks to the wider acceptance of DL for clinical applications. Even when the DL-based method shows good performance in many cases, it is difficult or almost impossible to explain how the networks perform various tasks. In recent years, the attention mechanism is explored to interpret DL Models, which attempts to build weights that reflect which part of the input is more important for decision making. 3) In real-world clinical applications, there are other issues including ethical, regulatory, and legal issues to solve, which should be carefully considered for the development of AI models in cancer imaging.

This Research Topic involved many wonderful works, which made full use of radiomics and AI in cancer imaging. We appreciate all the reviewers and authors for their contributions to this Research Topic, and we hope this Research Topic can gain more attention in the related fields.

## Author Contributions

The author confirms being the sole contributor of this work and has approved it for publication.

## Funding

This work was supported in part by the National Natural Science Foundation of China under Grant 42071302, the Innovation Program for Chongqing Overseas Returnees under Grant cx2019144.

## Conflict of Interest

The author declares that the research was conducted in the absence of any commercial or financial relationships that could be construed as a potential conflict of interest.

## Publisher’s Note

All claims expressed in this article are solely those of the authors and do not necessarily represent those of their affiliated organizations, or those of the publisher, the editors and the reviewers. Any product that may be evaluated in this article, or claim that may be made by its manufacturer, is not guaranteed or endorsed by the publisher.

